# Sugar-rich larval diet promotes lower adult pathogen load and higher survival after infection in a polyphagous fly

**DOI:** 10.1242/jeb.243910

**Published:** 2022-08-23

**Authors:** Hue Dinh, Ida Lundbäck, Sheemal Kumar, Anh The Than, Juliano Morimoto, Fleur Ponton

**Affiliations:** 1 School of Natural Sciences, Macquarie University, North Ryde, NSW 2109, Australia; 2 Department of Entomology, Vietnam National University of Agriculture, Trau Quy, Gia Lam, Hanoi 100000, Vietnam; 3 School of Biological Sciences, University of Aberdeen, Zoology Building, Tillydrone Ave, Aberdeen AB24 2TZ, UK; 4 Programa de Pós-graduação em Ecologia e Conservação, Universidade Federal do Paraná, Curitiba 82590-300, Brazil

**Keywords:** Fitness, Immunity, Macronutrient, Nutrition, Resistance

## Abstract

Nutrition is a central factor influencing immunity and resistance to infection, but the extent to which nutrition during development affects adult responses to infections is poorly understood. Our study investigated how the nutritional composition of the larval diet affects the survival, pathogen load and food intake of adult fruit flies, *Bactrocera tryoni*, after septic bacterial infection. We found a sex-specific effect of larval diet composition on survival post-infection: survival rate was higher and bacterial load was lower for infected females raised on a sugar-rich larval diet than for females raised on a protein-rich larval diet, an effect that was absent in males. Both males and females were heavier when fed a balanced larval diet compared with a protein- or sugar-rich diet, while body lipid reserves were higher for those that had consumed the sugar-rich larval diet compared with other diets. Body protein reserves were lower for flies that had been raised on the sugar-rich larval diet compared with other diets in males, but not females. Both females and males shifted their nutrient intake to ingest a sugar-rich diet when infected compared with sham-infected flies without any effect of the larval diet, suggesting that sugar-rich diets can be beneficial to fight off bacterial infection as shown in previous literature. Overall, our findings show that nutrition during early life can shape individual fitness in adulthood.

## Abstract (precis)

**Summary:** Adult females of a polyphagous fly fed a diet rich in sugar at the larval stage show increased resistance to infection, indicating developmental conditions influence adulthood.

## INTRODUCTION

Environmental conditions during development can influence many aspects of adult phenotype and fitness. In humans, under- or over-nutrition at the fetal stage can increase predisposition to metabolic disease at the adult stage (see review in Gluckman et al., 2008). In birds, when conditions are unfavourable during early development, growth and adult immunity are affected (Butler and McGraw, 2011; Naguib et al., 2006, 2004; Tella et al., 2001; Krause et al., 2009; but see also Råberg and Stjernman, 2003; Tschirren et al., 2009). In holometabolous insects, the nutritional resources acquired at the larval stage are crucial for survival during metamorphosis (Arrese and Soulages, 2010; Mirth and Riddiford, 2007). When faced with food restrictions and unbalanced diets at the larval stage, insects show delayed development (May et al., 2015; Zwaan et al., 1991; Telang et al., 2007; Colasurdo et al., 2009; Roeder and Behmer, 2014; Xie et al., 2015), lower adult body size (Zwaan et al., 1991; Colasurdo et al., 2009; Xie et al., 2015; Morimoto et al., 2017, 2019; Boggs and Freeman, 2005; Wang et al., 2016) and decreased lifespan (Bauerfeind and Fischer, 2009; Runagall-Mcnaull et al., 2015; but see May et al., 2015, where *Drosophila* adult life span was increased when fed a protein-restricted diet during the larval stage). Adult reproductive performance is also affected by larval diet restriction, with lower courtship levels, a lower number of matings, lower investment in reproductive organs and a decrease in the total number of offspring produced compared with individuals that were fed *ad libitum* at the larval stage (Colasurdo et al., 2009; Xie et al., 2015; Morimoto et al., 2017; Wang et al., 2016; Morimoto et al., 2016; Kaspi et al., 2002; Dmitriew and Rowe, 2011; Yan et al., 2021).

Nutrition during development affects not only life-history traits but also resistance to infection. In insects, food shortage at the larval stage strongly reduces immune activity, as observed in adult damselflies *Lestes viridis* (Rolff et al., 2017). Larval food quality (i.e. yeast-to-sugar ratio) also influences the expression of antimicrobial peptide genes (diptericin A, *DptA*; and metchnikowin, *Mtk*) in adult *Drosophila*, with an increase in expression when the yeast-to-sugar ratio in the larval diet was increased (Fellous and Lazzaro, 2010). In the cotton leafworm, *Spodoptera littoralis*, a change in the larval diet affects lytic and phenoloxidase activity without any evidence of a change in immune gene expression (Cotter et al., 2019). The effects of larval diet on immune-challenged adults have recently been described in female *Anopheles coluzzii*, with changes in the prevalence and intensity of *Plasmodium berghei* infection (Linenberg et al., 2016). However, it remains unclear which aspect of the larval diet (quality or quantity) induces differences in pathogen load and survival rate of the infected individuals. Interestingly, in hemimetabolous insects, Kelly et al. (2013) found a sex-specific effect of juvenile diet on the survival of adult crickets *Gryllus texensis* when infected with the pathogenic bacterium *Serratia marcescens*, using defined diets (i.e. low- or high-protein diets) (Kelly and Tawes, 2013). However, crickets were fed the same experimental diet at both juvenile and adult stages, and, therefore, it is difficult to distinguish between the effects of juvenile and adult diet on adult resistance in this system.

Studies on the effects of developmental diet on adult immunity and resistance to infection, especially when adults are immune challenged, are still scarce or have been only partial for three main reasons. First, diet manipulations during development have focused on the quantity of food available, and there have been very few studies investigating how diet composition might affect adult resistance. The nutritional environment is likely to vary not only in the quantity of food available but also in the quality of food sources with nutritional imbalances. Second, the nutritional requirements of immune responses can be different between sexes (Fanson et al., 2013). For instance, encapsulation ability increases with the intake of both protein and sugar in females of decorated crickets, whereas male encapsulation ability only increases with protein intake (Rapkin et al., 2018). Hence, it remains to be tested whether pathogen resistance in both adult males and females is affected similarly by juvenile diet. Third, individuals might compensate for unfavourable developmental nutritional conditions by modifying their diet at adult stage (Poças et al., 2020). Exploring the effects of variation in the quality of juvenile diet on adult nutritional responses and body energetic reserves would give us insight into the extent to which nutritional conditions early in life modulate adult physiology and fitness.

Here, we manipulated the ratio of macronutrients (yeast-to-sugar ratio, Y:S) in the larval diet of the holometabolous fruit fly *Bactrocera tryoni*. We investigated the effects of larval diet manipulation on (i) developmental traits (i.e. percentage egg hatching, percentage pupation, percentage emergence and developmental time); (ii) adult physiological traits (i.e. total body mass, body lipid and protein); and (iii) adult response to a septic infection with the pathogenic bacterium *S. marcescens* (i.e. bacterial load, survival and food intake). Our findings provide new insights into how environmental experience during the larval stage influences a broad range of life-history traits as well as the outcome of septic infection in adulthood.

## MATERIALS AND METHODS

### Fly stock

Eggs were collected from a laboratory-adapted stock of Qfly (>20 generations old). Fly stock was maintained on a gel-based diet (i.e. standard rearing diet) at larval stage (Dinh et al., 2019) and a 1:3 ratio of hydrolysed yeast (MP Biomedicals cat. no 02103304) to sugar (CSR^®^ White Sugar) (Y:S) was provided separately at the adult stage. Flies were reared in a controlled environment room under the conditions of 25°C and 65% humidity, with a 12 h light:dark cycle at Macquarie University (North Ryde, NSW, Australia). Eggs were collected from the fly stock colony for 2 h using an ovipositional device that consisted of a plastic bottle with numerous puncture holes, filled with 30 ml of water to maintain humidity. The collected eggs were used to assess the effects of developmental diet on development and adult traits.

### Diet preparation

Three larval diets varying in the Y:S ratio were prepared (listed in Table S1). The standard diet is considered optimized for larval development, and it has been routinely used to rear *B. tryoni* (Y:S 1.67:1) (Dinh et al., 2019). We manipulated the relative amount of yeast and sugar (Dinh et al., 2019) to generate unbalanced diets, including a ‘protein-rich diet’ (Y:S 5:1) and a ‘sugar-rich diet’ (Y:S 1:3.4). These diets have been found to modulate the development and adult life-history traits of *B. tryoni* flies (Moadeli et al., 2017). The yielding percentage of protein (w/w Y+S) in the three substrates was 70% for Y:S 5:1, 43% for Y:S 1.67:1 and 12% for Y:S 1:3.4. All ingredients were mixed into warm water and the final volume (250 ml) was achieved by adding distilled water. Citric acid was added to adjust the pH of the diet solution to 3.5 at room temperature. To assess developmental traits, diet plates were prepared by pouring 25 ml of larval gel diet into 100 mm plates. When we needed to rear a large number of larvae, 150 ml of diet was poured into plastic trays (17.5 cm long, 12 cm wide, 4 cm deep).

### Development traits

Groups of 100 eggs were transferred to a black filter paper previously soaked in distilled water and placed onto the diet plates. The plates were then covered with their lids and kept under controlled laboratory conditions during larval development. Nine replicates per larval diet treatment were performed simultaneously (i.e. nine plates). The number of unhatched eggs was counted 4 days post-seeding under a stereomicroscope. The black filter paper and unhatched eggs were then removed from the diet plates. The lids of the diet plates were opened 7 days post-seeding; plates were then placed on 50 ml of autoclaved fine vermiculite to allow larvae to jump outside the plates and pupate. The total number of pupae was then recorded for each plate, and pupae were placed into partially netted 12.5 l plastic cages for emergence (9 replicates). The number of pupae that did not emerge was recorded over 4 days.

### Adult traits

We seeded ∼600 eggs into 150 ml diet to achieve the same density as in the developmental experiment (100 eggs per 25 ml diet). To do so, we dispensed 40 µl of an egg solution at ∼150 eggs µl^−1^ (average value calculated for 6 replicates)*.* Eggs were allowed to develop until the adult stage. One-day-old adults (i.e. collected 1 day after eclosion) were used for the different measurements.

#### Adult dry body mass

Flies were collected and stored at −20°C. Carcasses were dried at 55°C for 48 h (Binder drying oven). Dry mass was measured using a microbalance (Sartorius, accuracy ±0.001 mg) for 30 individual flies of each sex per diet treatment.

#### Adult body lipid reserves

Body lipid reserves were extracted in three, 24 h changes of chloroform as previously described (Nguyen et al., 2019). At the end of the third chloroform wash, lipid-free bodies were re-dried and re-weighed to calculate lipid content. We performed 15 replicates (i.e. 15 individual flies of each sex) per diet treatment.

#### Adult body protein reserves

After lipid extraction, fly bodies were crushed in 300 µl 0.1 mol l^−1^ NaOH and centrifuged at 8000 rpm for 30 s; 100 µl of supernatant was collected in new Eppendorf tubes and diluted 1:10. We transferred 5 µl of the diluted solution to 96-well plates and allowed it to react with 200 µl of Bradford reagent (Sigma-Aldrich). Plates were incubated for 5 min at room temperature, and absorbance was measured at 595 nm using a spectrometer (Eppendorf). We ran 15 biological replicates (i.e. 15 individual flies of each sex) per diet treatment. Each sample was run in three technical replicates. The Bradford assay was calibrated using a standard curve generated from six different concentrations of IgG protein (Sigma-Aldrich) (0.2, 0.15, 0.1, 0.05, 0.025 and 0 µg µl^−1^).

### Bacterial infection

*Serratia marcescens* (ATCC 13880, Thermo Fisher Scientific) was inoculated into 5 ml of sterile Nutrient Broth (Oxoid, CM0001) and incubated overnight (approximately 16 h) at 26°C with shaking at 200 rpm. The bacterial culture was centrifuged at 10,000 ***g*** at 4°C for 2 min. The supernatant was discarded, and the bacterial pellet washed twice using 1× phosphate buffered saline (PBS; Sigma-Aldrich, cat. no P4417) to remove any trace of the medium. The bacterial pellet was resuspended to a target concentration of OD_600_=0.025 in sterile PBS.

One day after adult eclosion, flies were cold anaesthetized at −20°C for 2 min and placed on a Petri dish on an MK20 Dry Bath at −10°C. Injections were performed using a 10 µl syringe (NanoFil) connected to a microinjector (World Precision Instruments) with a delivery speed of 50 nl s^−1^. A volume of 0.2 µl of the bacterial solution, yielding a dose of approximately 1680 cells, was injected into the fly's coxa of the third right leg. PBS-injected (i.e. sham-treated) flies were used as controls.

### Bacterial load

Bacterial load was measured in infected flies, with females and males being individually crushed in 100 µl of PBS, followed by serial dilution to 1:10 and 1:100. A volume of 10 µl from each dilution was plated onto nutrient agar supplemented with 30 µg ml^−1^ tetracycline (Sigma-Aldrich) (Nguyen et al., 2019) and incubated at 26°C for 48 h. The number of bacteria on each plate was counted through counting the number of colony forming units (CFU). We measured the average concentration of CFU for each dilution. The bacterial load was measured 6, 24, 48 and 96 h post-infection (PI) (10 cages per diet). We sampled 1 fly per replicate cage (i.e. 10 individual flies of each sex) for each diet treatment at each time point. *Serratia marcescens* was not present in our fly stock. This was checked by crushing individual flies (12 individual flies of each sex) in 100 μl PBS, and 25 μl of the solution was plated onto nutrient agar supplemented with 30 μg ml^−1^ tetracycline, and incubated at 26°C for 48 h.

### Survival after infection

One day after adult eclosion, adult flies (males and females) were injected with either PBS or live bacteria (as above). Injected flies were then maintained in groups of 25 in 1.25 l cages (10 cm×10 cm×12.5 cm) and provided with food and water *ad libitum*. Dead flies were counted and removed daily from the cages. We initially limited the experimental time to 4 days PI, in which we measured bacterial load. The low mortality rate after 4 days PI led us to extend the time frame of the survival experiment until 15 days PI. We ran three replicates (three cages) per diet.

### Food intake

The method to measure and calculate food intake was previously described in Nguyen et al. (2019). Briefly, flies were housed individually and allowed to self-select between a sugar (CSR^®^ White Sugar) and a yeast (MP Biomedicals cat. no. 02103304) solution. Sugar and yeast were provided separately in two 30 µl capillaries at a final concentration of 160 g l^−1^. The hydrolysed yeast used in this study was the only source of protein available to the flies, containing approximately 62.1% protein and 1% sugar. Final macronutrient (i.e. protein and sugar) intake (µg) was calculated based on these values.

### Statistical analyses

Statistical analyses and graphing were performed using R (http://www.R-project.org/). We fitted generalized linear models (GLM) with quasibinomial distribution to analyse the proportion of egg hatching, pupation, emergence and body reserves. We fitted GLM with Gaussian distribution to analyse the dry body mass and bacterial load (log-transformed) of infected flies. Equality of variance and normal distribution were checked graphically. To analyse the survival data, we could not use a Cox regression because the proportional-hazards assumption was not met (*p* global=0.017). We therefore analysed the percentage of flies that died 4 and 15 days PI using GLM with a quasibinomial distribution. Because only a very small number of PBS-injected flies died during the course of the experiment (15 days), the analysis was performed only for infected flies across diets. Student–Newman–Keuls (SNK) *post hoc* tests with *P*<0.05 were applied to identify treatments that differ from each other.

## RESULTS

### Effects of larval diet on developmental traits

**Fig. 1.**
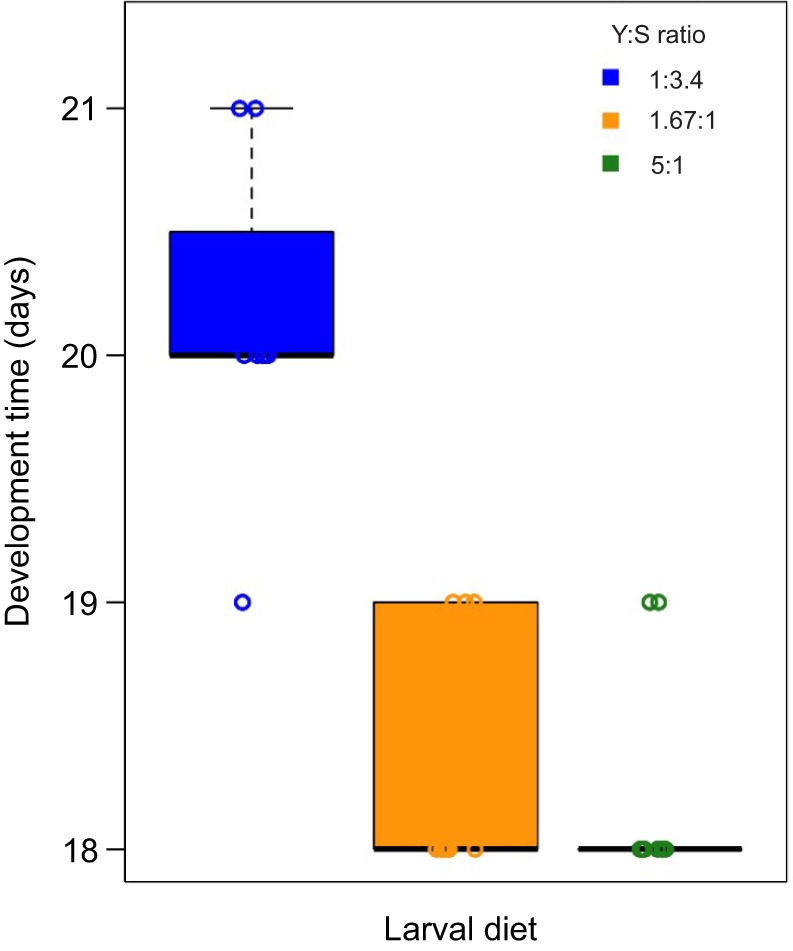
**Effect of larval diet on egg-to-adult developmental time.** Nine groups of 100 eggs were reared on three diets varying in the yeast-to-sugar ratio (Y:S ratio): 1:3.4 (sugar-rich larval diet; blue), Y:S 1.67:1 (balanced larval diet; orange) and Y:S 5:1 (protein-rich larval diet; green). Box plots show median, upper and lower quartiles and 1.5× the interquartile range; circles are data points.

Larval diet did not influence the percentage egg hatching (GLM, *F*_2,24_=0.01, *P*=0.905), the percentage pupation (GLM, *F*_2,23_=1.940, *P*=0.166) and the percentage emergence (GLM, *F*_2,23_=1.111, *P*=0.346). The percentage egg hatching and the percentage pupation were around 90% in all larval diet treatments. The percentage emergence was around 98% across the larval diets. However, larval diet had a significant effect on the egg-to-adult developmental time (Fig. 1) (GLM, *F*_2,23_=33.896, *P*<0.001). As expected, developmental time was longer for the larvae fed the sugar-rich larval diet (mean±s.e.m., 20.13±0.641 days) than for those fed the balanced (18.33±0.500 days) and protein-rich larval diets (18.22±0.441 days) (Fig. 1).

### Effects of larval diet on adult body mass, total body lipid and body protein

**Fig. 2.**
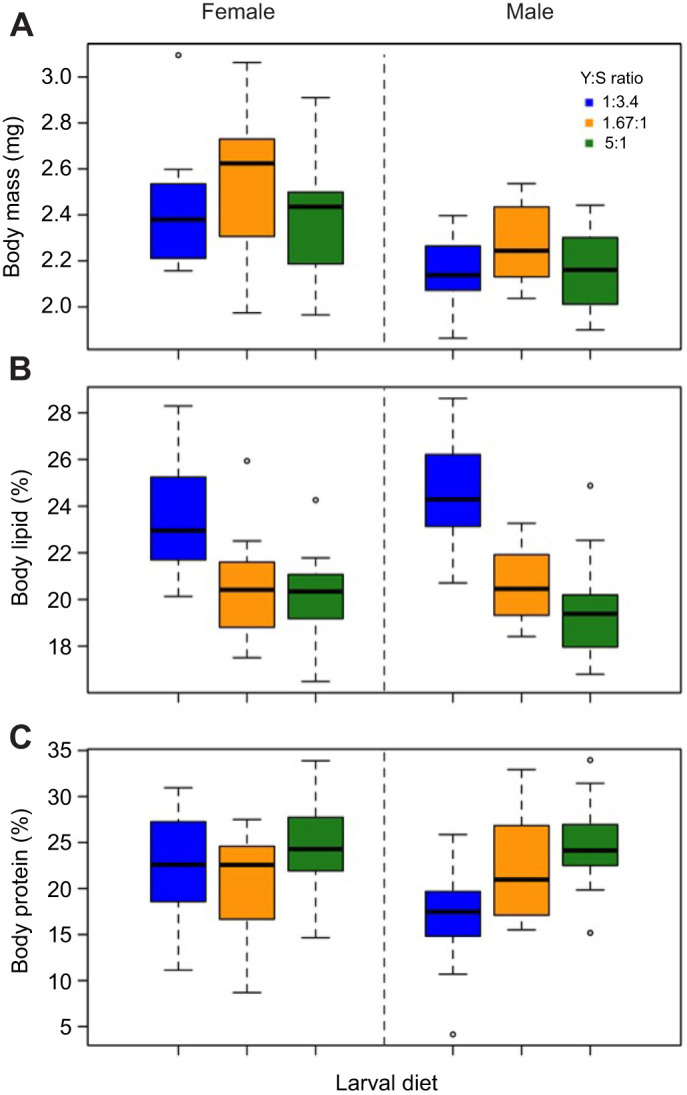
**Effect of larval diet on adult body mass, and lipid and protein body reserves.** Body mass (A), lipid body reserves (B) and protein body reserves (C) were measured in male and female flies fed three diets varying in Y:S ratio at the larval stage: Y:S 1:3.4 (sugar-rich larval diet, *N*=12 females, *N*=14 males; blue), Y:S 1.67:1 (balanced larval diet, *N*=15 females, *N*=15 males; orange) and Y:S 5:1 (protein-rich larval diet, *N*=14 females, *N*=15 males; green). Circles are outliers.

Dry body mass was significantly influenced by larval diet and sex (GLM; Sex: *F*_1,81_=32.59, *P*<0.001; Larval diet: *F*_2,82_=4.64, *P*=0.012; Larval diet×Sex: *F*_2,79_=3.73, *P*=0.078). Adults flies reared on the standard diet at larval stage had a higher body mass relative to those reared on either the protein-rich or the sugar-rich larval diet (Fig. 2). Additionally, adult body mass was higher in females than in males (Fig. 2). The percentage of body lipid reserves was significantly influenced by larval diet for both females and males (GLM; Sex: *F*_1,81_=0.111, *P*=0.739; Larval diet: *F*_2,82_=26.1868, *P*<0.001; Larval diet×Sex: *F*_2,79_=1.165, *P*=0.312). Body lipid reserves were greater in the adult flies from the sugar-rich larval diet group than in flies from the balanced and protein-rich diet groups (Fig. 2). However, we found that the interaction between sex and larval diet composition significantly influenced the percentage of body protein reserves (GLM; Sex: *F*_1,81_=25.522, *P*<0.001; Larval diet: *F*_2,82_=182.757, *P*<0.001; Larval diet×Sex: *F*_2,79_=14.928, *P*<0.001). The body protein reserve of males from the sugar-rich larval diet group was lower than that of males from the protein-rich and balanced larval diet groups (Fig. 2). We did not detect any effect of larval diet on females' body protein reserves (Fig. 2).

### Effects of larval diet on bacterial load of adult flies

**Fig. 3.**
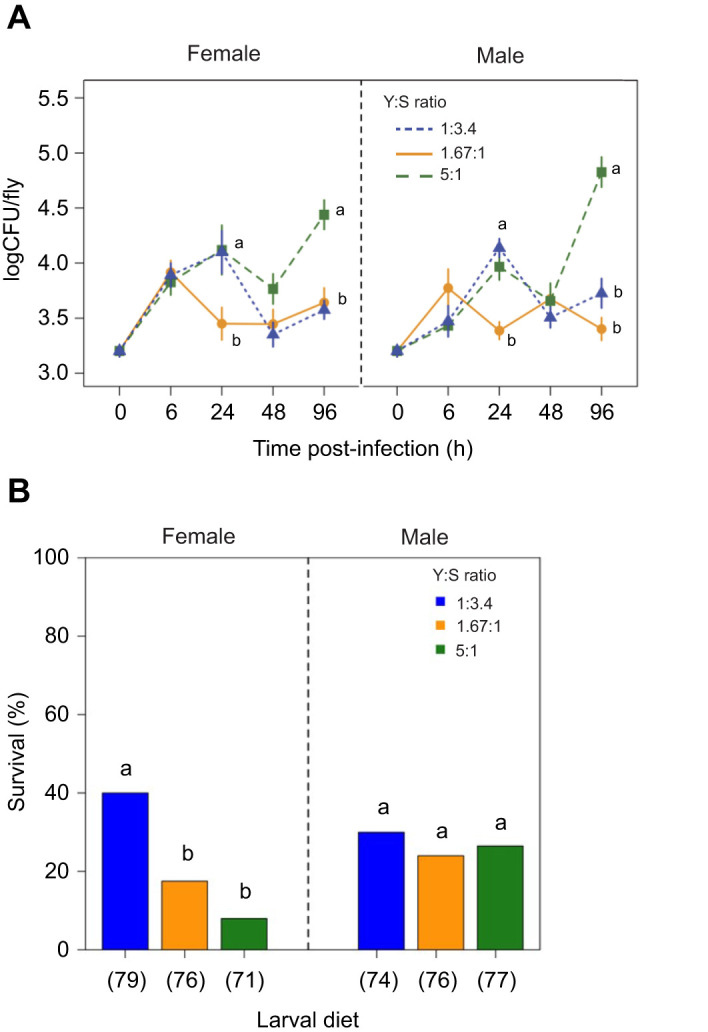
**Effect of larval diet on bacterial load and survival after infection.** (A) Effect of larval diet on bacterial load [number of colony-forming units (CFU) per fly] measured at 0, 6, 24, 48 and 96 h post-infection with *Serratia marcescens* for flies (*N*=10) fed three larval diets varying in Y:S ratio: 1:3.4 (sugar-rich larval diet; blue), Y:S 1.67:1 (balanced larval diet; orange) and Y:S 5:1 (protein-rich larval diet; green). Symbols indicate mean values (±s.e.m.). (B) Effects of larval diet on the survival rate of infected females and males at 15 days post-infection. Numbers in parentheses below the bars indicate the number of flies in each treatment. Different letters indicate significant differences between larval diets, assessed by SNK test at *P*<0.05.

The two-way interaction between larval diet and time influenced the bacterial load of infected flies (*P*<0.001; Table 1). Bacterial load was comparable between larval diets at 6 and 48 h PI (Fig. 3A). At 24 h PI, bacterial load tended to be higher in flies fed the protein- and sugar-rich larval diets relative to flies fed the balanced diet (Fig. 3A). At 96 h PI, the bacterial load of flies fed the protein-rich diet was greater than that of flies fed either the balanced or sugar-rich larval diet (Fig. 3A). We did not observe any significant effect of the two-way interaction between larval diet and sex (*P*=0.952; Table 1) and between sex and time (*P*=0.062; Table 1) on bacterial load. The three-way interaction between diet, sex and time was also not significant (*P*=0.155; Table 1).

**Table 1.**
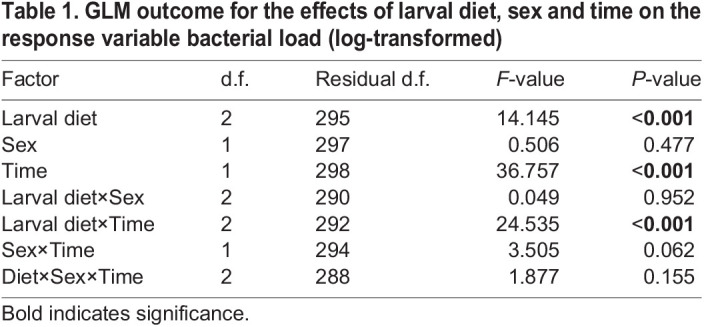
GLM outcome for the effects of larval diet, sex and time on the response variable bacterial load (log-transformed)

### Effects of larval diet on survival of infected flies

At 4 days PI, the survival of infected flies was not affected by larval diet or sex (Larval diet: *F*_2,450_=1.421, *P*=0.241; Sex: *F*_1,449_=0.291, *P*=0.589; Larval diet×Sex: *F*_2,447_=2.500, *P*=0.083). At 15 days PI, however, we observed a significant effect of the interaction between larval diet and sex (Larval diet: *F*_2,450_=2.949, *P*=0.053; Sex: *F*_1,449_=0.098, *P*=0.754; Larval diet×Sex: *F*_2,447_=4.600, *P*=0.010). Survival of infected females from the sugar-rich larval diet group was significantly higher than that of females from the balanced and protein-rich larval diet groups (Fig. 3B); however, we did not detect any effects of larval diet on the survival of infected males (Fig. 3B).

### Effects of larval diet on the nutritional choice of immune-challenged adult male and female flies

**Fig. 4.**
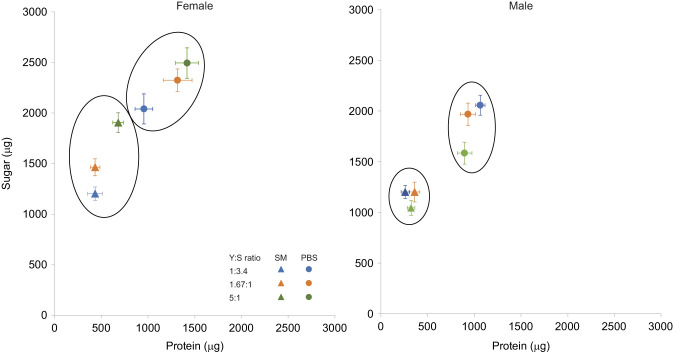
**Total macronutrient intake.** Macronutrient intake was measured for 4 consecutive days for female and male flies reared on three larval diets varying in Y:S ratio, following injection with either *Serratia marcescens* (SM, triangles) or PBS (control; circles): Y:S 1:3.4 (sugar-rich larval diet, *N*=12 female SM, *N*=12 female PBS, *N*=9 male SM, *N*=9 male PBS; blue), Y:S 1.67:1 (balanced larval diet, *N*=14 female SM, *N*=12 female PBS, *N*=19 male SM, *N*=12 male PBS; orange) and Y:S 5:1 (protein-rich larval diet, *N*=19 female SM, *N*=12 female PBS, *N*=15 male SM, *N*=12 male PBS; green). Symbols indicate mean (±s.e.m.) protein (horizontal) and sugar (vertical) cumulative intake.

The ingested macronutrient ratio (protein-to-sugar ratio, P:S ratio) was influenced by treatment and sex (*P*<0.001; Table 2). Infected flies ingested a lower P:S ratio [i.e. diet richer in sugar, P:S ∼0.302 (1:3.3)] compared with PBS-injected flies [i.e., P:S ∼0.537 (1:1.8)] (Fig. 3). Females ingested a diet that was slightly richer in protein than that ingested by males [P:S females ∼0.430±0.203 (1:2.3); P:S males ∼0.373±0.180 (1:2.7)]. The amount of protein ingested after 4 days was influenced by the interaction between larval diet and injection treatment (*P*=0.021; Table 2). There was a trend for flies from the sugar-rich larval diet group to ingest less protein than the individuals from the two other larval diet groups (Fig. 4). This trend was more marked in PBS-injected individuals than in bacteria-injected ones, because infected individuals ingested less food (Fig. 4). The total quantity of sugar ingested after 4 days was slightly influenced by the interaction between larval diet and sex (*P*=0.050; Table 2). As observed for protein intake, individuals from the sugar-rich larval diet tended to ingest less sugar (Fig. 4). In females, sugar intake tended to increase with the protein content of the larval diet (Fig. 4); in particular, infected females reared on the protein-rich diet ingested the highest quantity of sugar (Fig. 4). This trend was also observed in males but was less clear (Fig. 4). Males tended to ingest less food than females (Fig. 4).

**Table 2.**
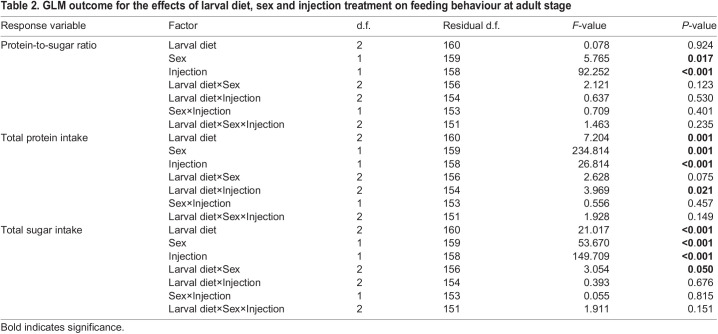
GLM outcome for the effects of larval diet, sex and injection treatment on feeding behaviour at adult stage

## DISCUSSION

We examined the effects of the macronutrient composition of larval diet on adult resistance to infection as well as on some developmental and physiological traits. When adult flies were challenged with *S. marcescens*, we observed a higher bacterial load in both males and females fed a protein-rich larval diet; however, only females showed a lower survival rate after infection. This might be partly explained by the finding that body lipid reserves were greater in adult flies from the sugar-rich larval diet group than in those from the balanced and protein-rich diet groups. The body protein reserves of males from the sugar-rich larval diet were also lower than those of males from the protein-rich and balanced larval diets. However, there was no effect of the larval diet on females' body protein reserves. The larval diet also influenced adult feeding choice, with flies from the sugar-rich larval diet group ingesting slightly less protein than the individuals from the two other larval diet groups.

### Larval diet influences adult pathogen resistance and macronutrient intake following infection with the bacterium *S. marcescens*

The bacterial load of infected flies was influenced by the nutritional conditions experienced at the larval stage. This might be for two reasons. First, pathogens require energy for growing, and thus the allocation of within-host energy reserves is essential to this process (Cressler et al., 2014; Smith, 2007; Bedhomme et al., 2004; Pulkkinen and Ebert, 2004; Hall et al., 2009). Here, we found that the total body reserves of protein and/or lipid were modulated by larval diet. Second, early-life nutrition affects host immune responses at later developmental stages, which might modulate the number of pathogenic cells in the host. In mosquitoes and *Drosophila*, poor larval conditions (i.e. starvation or protein restriction) have been shown to alter the expression of adult immune-related genes (Fellous and Lazzaro, 2010; Muturi et al., 2011).

Despite differences in the bacterial load between larval diet treatments, the survival of infected flies was similar during the first 4 days PI. Infected flies might only start dying when the number of pathogenic bacteria reaches a certain level defined as ‘bacterial load upon death’ (Duneau et al., 2017), and this level may not have been reached only a few days after infection. At 15 days PI, however, infected females that were reared on the sugar-rich larval diet survived at a greater rate compared with those kept on the protein-rich larval diet. Several hypotheses can explain these results. First, a lower bacterial load at the early stage of infection might have slowed down the time required to reach the ‘bacterial load upon death’ in the infected females fed the sugar-rich larval diet. Second, it has been previously shown that infected flies reduce total food intake and shift diet choice towards a sugar-rich diet which promotes their survival after infection (Nguyen et al., 2019). We also found that infected adult flies ingested a lower P:S ratio compared with PBS-injected flies (similar result to that in Ponton et al., 2020). Further, flies from the sugar-rich larval diet group tended to ingest less protein than individuals from the two other larval diet groups, which might provide them with better resistance to infection for the positive effects of anorexia on host defence. Third, the female flies raised on the sugar-rich larval diet might have invested more in immunity at the expense of other life-history traits. This is supported by studies in birds and insects showing the negative correlation between immune function and developmental time (Boots and Begon, 1993; Rantala and Roff, 2005). For instance, female moths reared on a sugar-rich larval diet allocate a lower proportion of their mass to the development of their ovaries (i.e. invest less in reproduction) compared with those on protein-rich diets (Colasurdo et al., 2009). Hence, female flies fed the sugar-rich larval diet potentially prioritize their immunity over developmental time (see also Stefana et al., 2017, for a similar discussion). Also, fat content serves as a crude estimate of the size of the fat body, the major immune responsive tissue in insects (Hoshizaki, 2012). Because body lipid reserves were higher in flies raised on the sugar-rich larval diet, it is possible that their immune system was more efficient at fighting the infection. Further explorations of the immune status, reproductive output and longevity of females raised on the different larval diets would provide insight into the effects of the juvenile nutritional environment on resource allocation and potential trade-offs between immune traits and other life-history traits.

Early-life environment, including nutrition, can be used to predict future adult environment, and individuals can develop proper behaviours to respond to environmental challenges in later developmental stages (Langenhof and Komdeur, 2018). The higher survival rate of the infected females raised on a sugar-rich larval diet might suggest that an unbalanced diet at the larval stage can act as a cue for higher disease risk in adulthood, and flies on this diet might invest more in defence (see also Linenberg et al., 2016; Kelly and Tawes, 2013; Boots and Roberts, 2012; Ben-Ami et al., 2010; Mitchell and Read, 2005). This does not, however, explain the sex-dependent effects in our results.

Unlike what we observed in female flies, the survival rates of infected adult males were comparable between the larval diet treatments despite a difference in bacterial load. While it is difficult at this stage to explain why the effect of larval diet on survival rate is sex specific, previous studies have shown that the diet composition can influence immunity differently in males and females. For instance, in fruit flies and crickets, while both protein and sugar intake affect phenoloxidase (PO) activity and encapsulation ability in females, only protein intake influences these immune traits in males (Fanson et al., 2013; Rapkin et al., 2018). Also, the magnitude of the effects of larval diet composition on PO activity and nitric oxide production in adults can be different between male and female mosquitoes, *Aedes aegypti*, with a stronger effect in females (Moreno-García et al., 2010). In parallel, larval diet can differently influence other adult traits in both sexes. In the butterfly *Melitaea cinxia*, larval food stress negatively affects the reproductive output of females, but not males (Rosa and Saastamoinen, 2017). Also, developing on a high yeast diet only benefits the life span of female *Drosophila* (Duxbury and Chapman, 2020). While there is evidence of sex-specific effects of diet on adult traits, the physiological mechanisms that are the basis of these differences remain to be investigated.

### Effect of larval diet on development traits

The percentage pupation and percentage emergence were similar between flies from the different larval diet treatments, suggesting that the unbalanced larval diets chosen here did not affect larval ability to survive metamorphosis. However, we found a significant effect of larval diet on egg-to-adult development time, and interestingly, this was only observed in the larvae fed the sugar-rich diet. This result is in accordance with previous observations in the forest tent caterpillar, *Malacosoma disstria* (Colasurdo et al., 2009; Despland and Noseworthy, 2006). The negative effect of the sugar-rich larval diet on development time is likely caused by the low protein level. Insect growth and metamorphosis are controlled by the insulin/target of rapamycin (TOR) signalling pathways (Sim and Denlinger, 2013; Mizoguchi and Okamoto, 2013; Nässel et al., 2015), which are triggered by high levels of amino acids (Zoncu et al., 2011; Chantranupong et al., 2015). Indeed, inhibition of the amino acid transporter gene has been shown to result in a lengthened development time (Fu et al., 2015)*.* Measuring the insulin/TOR activity in larvae fed the experimental diets would give insight into their metabolic state and deepen our understanding of the links between low-protein feeding and delayed developmental time.

### Conclusion

The present study highlights the sex-specific effects of larval diet composition on the survival of adult fruit flies after infection. Protein-rich larval diet promoted higher bacterial load and lower survival in female flies. The profound effects of larval diet on the developmental and physiological traits of adults were also demonstrated. A better understanding of the carry-over effects of the environmental conditions experienced in early life on life-history traits of individuals and population dynamics is a central goal in ecology (Sutherland et al., 2013). This will further assist the protection of endangered species, especially in the context of dramatic environmental changes that potentially lead to decreases in food availability and changes in food composition, as well as the introduction of infectious diseases to wildlife populations (Acevedo-Whitehouse and Duffus, 2009; Deem et al., 2001).

## Supplementary Material

Supplementary information
