# Supplementary material for: Sugar-rich larval diet promotes lower adult pathogen load and higher survival after infection in a polyphagous fly
Source: J Exp Biol. 2022 Aug 23;225(16):jeb243910. doi: 10.1242/jeb.243910 (PMC9482150; doi:10.1242/jeb.243910)
Supplement: Supplementary information [file jexbio-225-243910-s1.pdf]

Table S1. Ingredients of the larval diets

Ingredient	Larval diet		
	YS 5:1 (protein-biased)	YS 1.67:1 (balanced)	YS 1:3.4 (sugar-biased)
Brewer yeast (g)	58.33	43.8	15.91
Sugar (g)	11.67	26.2	54.09
Agar (g)	2.5	2.5	2.5
Nipagin (g)	0.375	0.375	0.375
Sodium benzoate (g)	0.375	0.375	0.375
Wheat Germ oil (μl)	375	375	375
Water	Final to 250 ml		
pH	Adjust to pH of 3.5 using Citric acid		

Journal of Experimental Biology • Supplementary information
